# The complete chloroplast genome sequence and phylogenetic analysis of *Volkameria inermis* Linnaeus 1753 (Lamiaceae), a tropical and subtropical coastal shrub

**DOI:** 10.1080/23802359.2025.2541623

**Published:** 2025-08-05

**Authors:** Mingzhong Liu, Wen Tang, Hui Zhang, Jiaxin Wu, Muqiu Zhao, Yunfeng Shi

**Affiliations:** ^a^ Yazhou Bay Innovation Institute of Hainan Tropical Ocean University, Sanya, China; ^b^ Key Laboratory for Coastal Marine Eco-Environment Process and Carbon Sink of Hainan province/Hainan Modern Marine Ranching Engineering Research Center, Hainan Tropical Ocean University, Sanya, China

**Keywords:** *Volkameria inermis*, chloroplast genome, clerodendrum, phylogenetic analysis, shrub

## Abstract

The complete chloroplast genome of *Volkameria inermis* Linnaeus 1753 (Lamiaceae) was sequenced and characterized for the first time. The chloroplast genome of *V. inermis* is 151,066 bp in length and comprises a large single-copy region (82,508 bp), a small single-copy region (17,302 bp), and a pair of inverted repeat regions each 25,628 bp. The genome encodes 87 protein-coding genes, 37 transfer RNA (tRNA) genes, and 8 ribosomal RNA (rRNA) genes. Phylogenetic analysis indicated that *V. inermis* clustered with 11 other species of the Volkameria genus. Among them, *Clerodendrum thomsoniae* was found to share the closest relationship with *V. inermis*.

## Introduction

*Volkameria inermis* Linnaeus 1753 (synonym: *Clerodendrum inermis* (Linnaeus) Gaertn. 1788) is a member of the Lamiaceae family within the order Lamiales. The Lamiaceae family includes 232 genera, many known for their aromatic properties (https://www.worldfloraonline.org/). The genus *Volkameria* (synonym: *Clerodendrum*) comprises over 500 species of small trees, shrubs, and herbs, many of which hold medicinal importance in Asia (Shrivastava and Patel 2007). *V. inermis* is one such species (Sundari and Shanthi 2022). This evergreen shrub primarily inhabits sandy coastal environments (El Mokni et al. 2013) and is characterized by pubescent branches, ovate-lanceolate leaves, white corollas with exserted stamens, and obovoid drupes measuring 6–11 mm in diameter. Its distribution spans tropical and subtropical Asia to the Western Pacific (https://www.worldfloraonline.org/). In China, *V. inermis* is found along the eastern and southern coastal regions and is often used for soil erosion control (Xiong et al. 2019). Despite its ecological and medicinal significance (Srisook et al. 2015), the chloroplast genome sequence of *V. inermis* has remained unavailable until now. This study presents the first complete chloroplast genome sequence of *V. inermis* and includes a phylogenetic analysis of its relationship with other species in the Volkameria genus. Our work aims to address this gap in chloroplast genomics and to provide insights into the evolutionary dynamics of the *Volkameria* genus.

## Materials and methods

**Figure 1.**
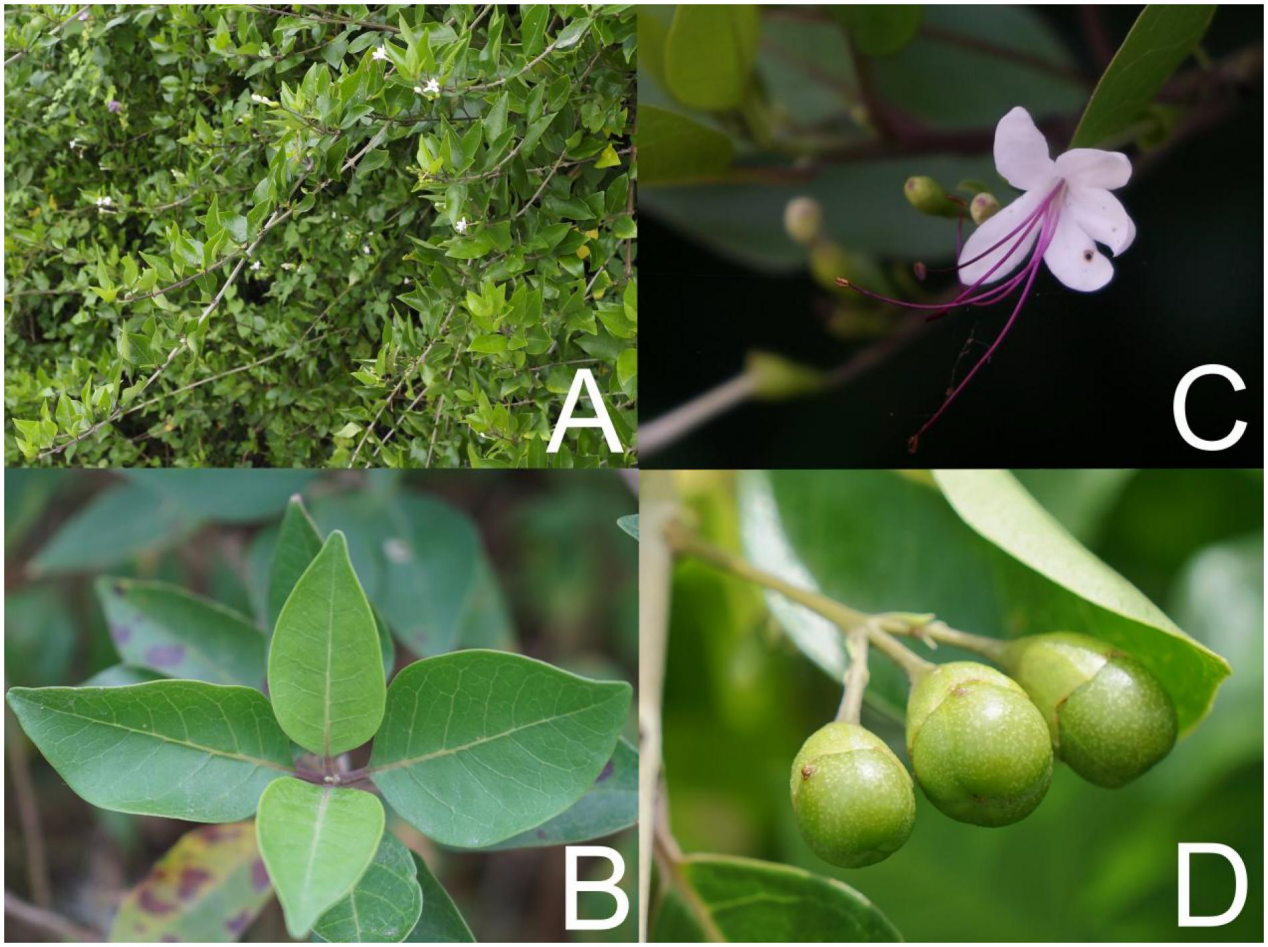
Photographic documentation of *Volkameria inermis* (Photo credit: Mingzhong Liu). (A) Whole Plant. A shrub with glabrous stems. (B) Leaves. Arranged oppositely and decussate, the leaf blades are ovate (3–12 cm long and 1.6–9 cm wide), glabrous, slightly fleshy, and penninerved. (C) Inflorescence and flowers. Flowers are arranged in terminal inflorescences, with 3–6 flowers per cluster. The flowers are actinomorphic and glabrous, featuring a white corolla (sometimes tinged with purple or pink) with a 2.3–3 cm long tube and exserted stamens measuring 2–5 cm. (D) Fruits. Subglobose to obovoid in shape, with a diameter of 6–11 mm.

Fresh leaves of *V. inermis* were collected from a shrub (Figure 1) on the shore of Xiaodonghai Bay (18°12′31.478″N; 109°30′23.810″E), southern Hainan Island, China. The area has a tropical oceanic monsoon climate, with an average annual temperature of 25.4 °C and approximately 2,563 h of sunshine per year (Chen et al. 2022). The specimen was deposited in the herbarium at the Laboratory of Seagrass Ecology and Carbon Sequestration, Hainan Tropical Ocean University (Contact: Yunfeng Shi, yunfengshi2025@163.com) under voucher number TOUSECO-00027.

Chloroplast genomic DNA was extracted using a Magnetic Plant Genomic DNA Kit (DP342; TIANGEN_BIOTECH, China). Genomic libraries were prepared with the NEBNext Ultra II DNA Library Prep kit (E7645L, New England Biolabs^®^ Inc.), and 150 bp paired-end sequencing was conducted on an Illumina Novaseq 6000 platform (Illumina, San Diego, CA). Raw reads were quality-checked using FastQC v0.12.0 (http://www.bioinformatics.babraham.ac.uk/projects/fastqc/), and the genome was assembled *de novo* with GetOrganelle v1.7.7.0 (Jin et al. 2020). The complete chloroplast genome of *V. inermis* was submitted to GenBank (accession number PQ463296.1). A circular genome map was generated using OGDraw v1.3.1 (Greiner et al. 2019), and maps for cis-splicing and trans-splicing genes were created with CPGview (http://www.1kmpg.cn/cpgview/). For phylogenetic analysis, 29 chloroplast genome sequences from related species and *Zingiber officinale* (Zingiberaceae) as the outgroup were obtained from GenBank. Sequences were aligned using MAFFT v7, and the phylogenetic tree was constructed with IQ-TREE v1.6.8 (model: GTR + I + G) (Nguyen et al. 2015) using 1,000 bootstrap replicates.

## Results

**Figure 2.**
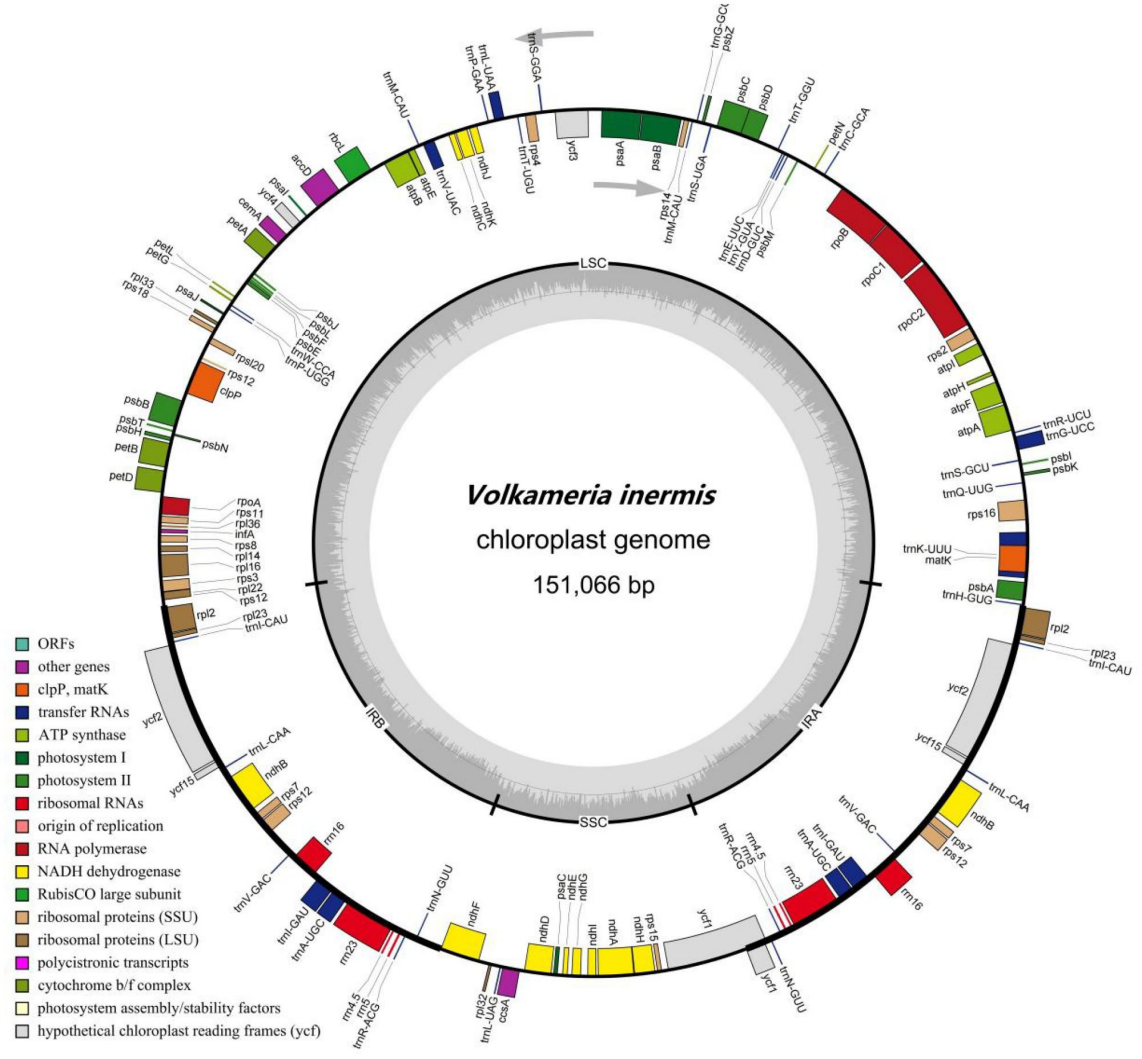
Schematic map of the chloroplast genome of *Volkameria inermis*, generated using OGDraw v1.3.1. The genome displays a typical circular structure with four distinct regions: a large single-copy (LSC) region, a small single-copy (SSC) region, and a pair of inverted repeats (IRa and IRb). Genes are color-coded based on their functional groups. On the outer circle, genes located outside the circle are transcribed counterclockwise, while those inside are transcribed clockwise. In the innermost circle, the darker gray shading indicates GC content, and the lighter gray represents AT content.

A total of 73,116,684 raw reads were generated, with 97.67% of bases at Q20 and 93.81% at Q30. After quality control, 71,419,664 clean reads were retained. The assembled chloroplast genome of *V. inermis* is 151,066 bp long, with an average sequencing depth of 7,449.8× and minimum depth of 90× (Figure S1). The genome exhibits a quadripartite structure comprising a large single-copy (LSC) region of 82,508 bp, a small single-copy (SSC) region of 17,302 bp, and two inverted repeat regions (IRA and IRB) of 25,628 bp each. The overall GC content is 38.3%, with 36.4% in the LSC, 32.1% in the SSC, and 43.3% in the IR regions. The genome contains 132 annotated genes: 86 protein-coding genes (PCGs), 37 tRNA genes, 8 rRNA genes, and 1 pseudogene. All tRNA genes exhibit cloverleaf secondary structure. Eight PCGs (*ndhB*, *petB*, *petD*, *atpF*, *rpl2*, *rpl16*, *rps16*, and *rpoC1*) and six tRNA genes (*trnA-UGC*, *trnG-UCC*, *trnI-GAU*, *trnK-UUU*, *trnL-UAA*, and *trnV-UAC*) each contain one intron, while two PCGs (*clpP*, *ycf3*) have two introns. Eight PCGs (*ndhB*, *rpl2*, *rpl23*, *rps12*, *rps7*, *rps19*, *ycf2*, and *ycf15*), seven tRNA genes (*trnA-UGC*, *trnI-CAU*, *trnL-CAA*, *trnN-GUU*, *trnR-ACG*, *trnV-GAC*, and *trnI-GAU*), and all rRNA genes are duplicated in the IR regions, while the pseudogene ycf1 occurs once (Figure 2). The structures of 13 cis-splicing PCGs (*rps16*, *atpF*, *rpoC1*, *ycf3*, *clpP*, *petB*, *petD*, r*pl16*, *rpl2*, *ndhB*, *ndhA*, *ndhB*, and *rpl2*) and one trans-splicing gene (*rps12*) are shown in Figures S2 and S3, respectively.

**Figure 3.**
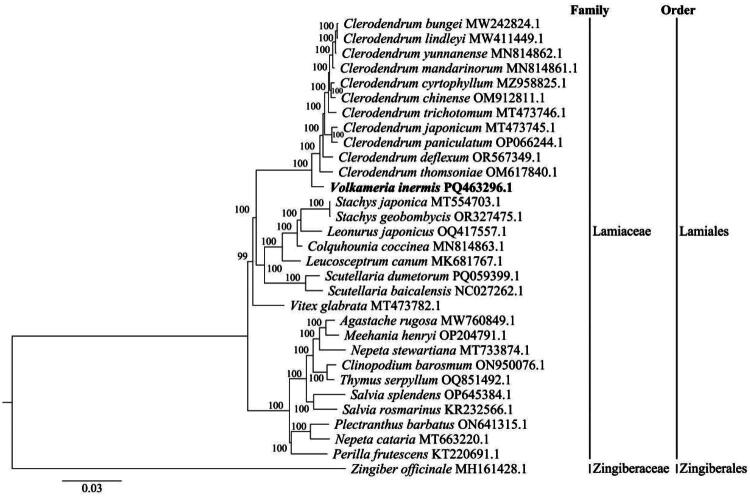
Maximum likelihood (ML) phylogenetic tree showing the position of *Volkameria inermis* (PQ463296.1) among 29 related species, constructed using complete chloroplast genome sequences. *Zingiber officinale* (MH161428.1) was designated as the outgroup. Numbers at the nodes represent bootstrap support values based on 1000 replicates. The GenBank accession numbers for the sequences used are as follows: MW242824.1 (Wang et al. 2021), MW411449.1 (Wan et al. 2022), MN814862.1 (Chen et al. 2021), MN814861.1 (Long et al. 2021), MZ958825.1 (Su et al. 2024), OM912811.1 (Chen et al. 2023), MT473746.1 (Ateto et al. 2024), MT473745.1 (Kamra et al. 2023), OP066244.1 (Chen et al. 2023), OR567349.1, OM617840.1 (Ateto et al. 2024), MT554703.1 (Wang et al. 2020), OR327475.1 (Wang et al. 2024), OQ417557.1 (Wang et al. 2023), MN814863.1 (Hämälä and Tiffin 2020), MK681767.1 (Chen et al. 2019), PQ059399.1 (Wang et al. 2024), NC027262.1 (Jiang et al. 2023), MT473782.1 (Zhao et al. 2021), MW760849.1 (Zhao and Yu 2023), OP204791.1, MT733874.1 (Hao et al. 2023), ON950076.1 (Lian et al. 2022), OQ851492.1, OP645384.1, KR232566.1 (Xia and Wen 2018), ON641315.1 (Kirankumar et al. 2023), MT663220.1 (Zhou et al. 2020), and KT220691.1 (Wen et al. 2023).

Phylogenetic analysis of complete chloroplast genomes places *V. inermis* within a well-supported clade of 11 other *Clerodendrum* species (Figure 3). The phylogenetic tree places *V. inermis* within the Lamiaceae family, where it forms a highly supported monophyletic clade with the genus *Clerodendrum* (100 bootstrap value). *V. inermis* is positioned as a sister taxon to the *Clerodendrum* species, such as *C. bungei* and *C. thomsoniae.*

## Discussion and conclusion

Taxonomic studies on *Clerodendrum* have revealed three distinct clades: African, Asian, and Pantropical Coastal. The Asian and African clades are sister groups, together forming a monophyletic lineage. It has been proposed to revive *Volkameria* for the Pantropical Coastal clade and restrict *Clerodendrum* to the African and Asian clades (Yuan et al. 2010). Similarly, another study suggested that *Clerodendrum* is monophyletic only when excluding certain tropical coastal species, which should be placed in a clade with *Volkameria* (Satthaphorn et al. 2024). Phylogenetic analysis places *V. inermis* within a clade of other *Volkameria* species (Long et al. 2021), where it occupies a relatively independent position. This suggests that *V. inermis* either diverged early from other *Volkameria* species or possesses unique genetic characteristics that distinguish it within the clade. This phylogenetic independence is further supported by its unique ecological adaptation to coastal environments, a trait not shared by other primarily inland *Volkameria* species (Wang et al. 2018; Kar et al. 2014; https://powo.science.kew.org/). Furthermore, a transcriptome analysis of *V. inermis* roots under 400 mM NaCl stress identified 98,968 unigenes, highlighting significant changes in plant hormone signaling genes that enhance salt tolerance (Xiong et al. 2019). These genetic adaptations likely underpin its ability to thrive in coastal habitats, setting it apart from its inland relatives. This study reports the first complete chloroplast genome of *V. inermis*, which exhibits a typical quadripartite structure of 151,066 bp. Phylogenetic analysis based on this chloroplast genome data confirms that *V. inermis* is closely related to the *Clerodendrum* genus within the Lamiaceae family. The resulting tree robustly supports its placement in this specific clade, clearly distinguishing it from other genera like *Stachys*, *Salvia*, or *Vitex*. These findings clarify the phylogenetic position of *V. inermis*, underscoring its close evolutionary relationship with *Clerodendrum*. Future studies exploring the ecological and genomic adaptations of *V. inermis* could provide deeper insights into the evolutionary dynamics of the *Volkameria* genus and its interactions with other plant groups.

## Supplementary Material

Supplementary Figures for Volkameria inermis.pdf

## Data Availability

The data that support the finding of this study are openly available inGenBank of NCBl at https://www.ncbi.nlm.nih.gov, reference number PQ463296.1 for *V. inermis*. The associated BioProject, BioSample, and SRA numbers are PRJNA1188740, SAMN44847880, and SRR31419613 respectively.
