# Supplementary material for: The complete chloroplast genome sequence and phylogenetic analysis of Volkameria inermis Linnaeus 1753 (Lamiaceae), a tropical and subtropical coastal shrub
Source: Mitochondrial DNA B Resour. 2025 Aug 5;10(9):779–83. doi: 10.1080/23802359.2025.2541623 (PMC12326380; doi:10.1080/23802359.2025.2541623)
Supplement: Supplementary Figures for Volkameria inermis.pdf [file TMDN_A_2541623_SM2600.pdf]

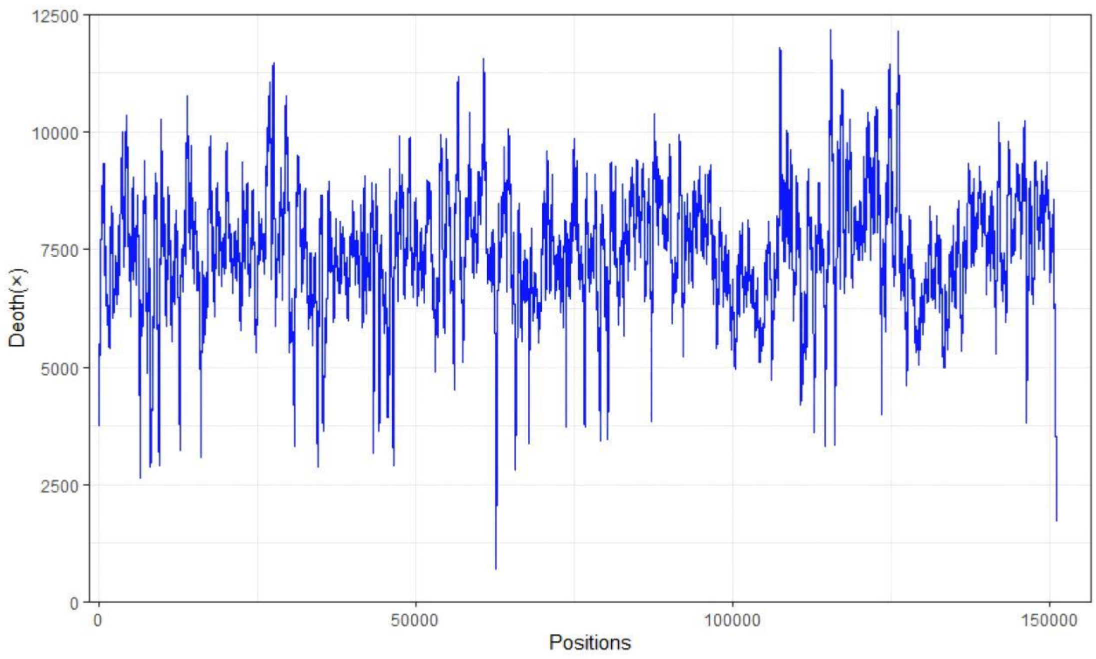


**Figure S1.** The map of sequencing depth, representing the sequencing depth on the complete chloroplast genome. The maximum sequencing depth is 12179×, the minimum sequencing depth is 90×, and the average sequencing depth is 7449.8×.

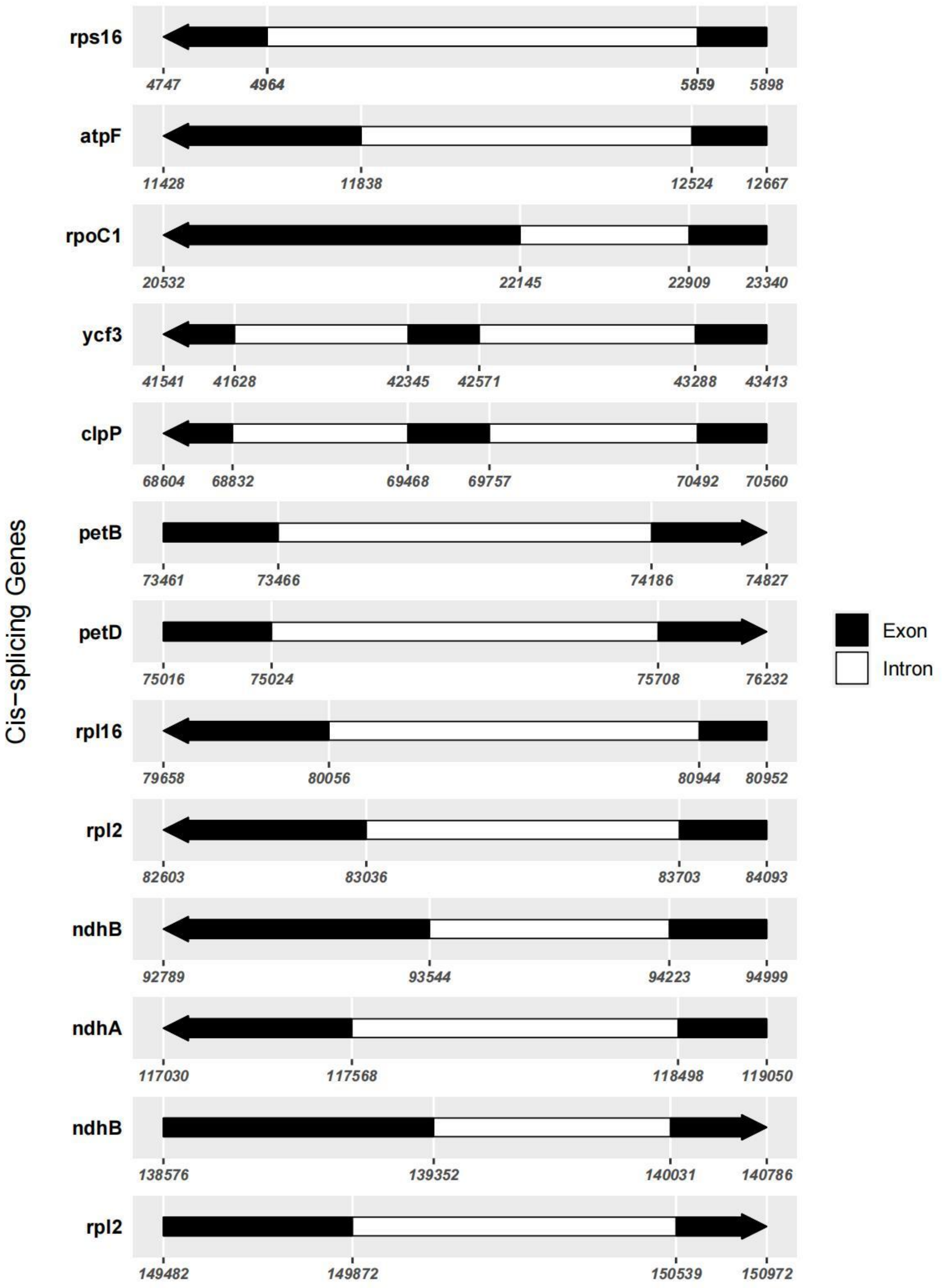


**Figure S2.** The map of the cis-splicing genes.

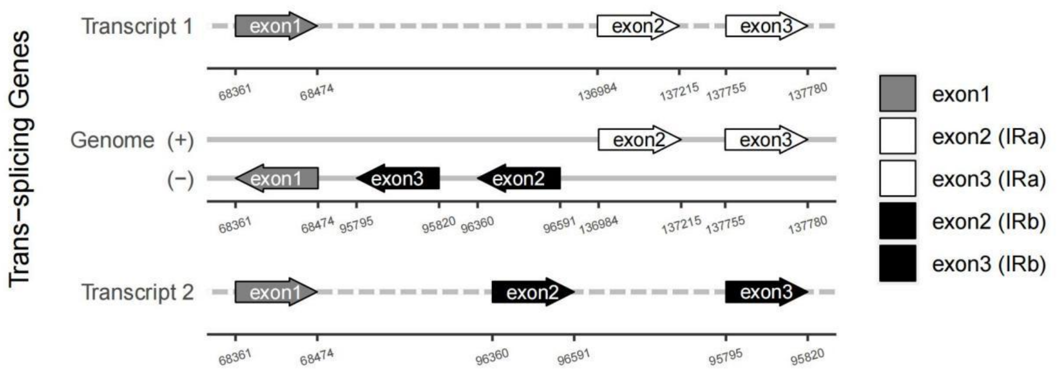


**Figure S3.** The map of the trans-splicing gene (*rps12*) on the chloroplast genome.
